# NLRP3 Inflammasome and Polycystic Ovary Syndrome (PCOS): A Novel Profile in Adipose Tissue

**DOI:** 10.3390/ijms27020699

**Published:** 2026-01-09

**Authors:** Salih Atalah Alenezi, Khalid Alshammari, Raheela Khan, Saad Amer

**Affiliations:** 1 Division of Translational Medical Sciences, School of Medicine, Royal Derby Hospital Centre, University of Nottingham, Derby DE22 3DT, UK; salih.alenezi@nottingham.ac.uk (S.A.A.); raheela.khan@nottingham.ac.uk (R.K.); 2 Prince Mohammed Bin Abdulaziz Medical City, Ministry of Health, Riyadh 14214, Saudi Arabia; 3 Nottingham Breast Cancer Research Centre, School of Medicine, Biodiscovery Institute, University of Nottingham, Nottingham NG7 2RD, UK; alyka16@nottingham.ac.uk

**Keywords:** PCOS, adipose tissue, NLRP3 inflammasome, IL-1β, CASP-1, PYCARD

## Abstract

Polycystic ovary syndrome (PCOS) is a common endocrine disorder characterized by chronic low-grade inflammation. The NLRP3 inflammasome has been implicated in various inflammatory conditions, but its role in PCOS remains unclear. This study aimed to investigate whether the NLRP3 inflammasome and its associated components, IL-1β, CASP-1, and PYCARD, are involved in the pathogenesis of PCOS. Gene and protein expression levels of NLRP3, IL-1β, CASP-1, and PYCARD were assessed in adipose tissue samples (visceral and subcutaneous) from women with and without PCOS using qPCR and Western blotting. Contrary to our initial hypothesis, CASP-1 gene expression was significantly higher in non-PCOS participants across all adipose depots examined. Similarly, NLRP3 protein levels were significantly upregulated in visceral adipose tissue (VAT) and in combined adipose samples from the non-PCOS group. No significant group differences were observed in the gene expression of NLRP3, IL-1β, or PYCARD. These findings suggest a more complex role for the NLRP3 inflammasome in PCOS than previously assumed. The elevated CASP-1 and NLRP3 levels in non-PCOS participants may reflect compensatory regulation, subclinical inflammation in controls, or technical variability. Further research is needed to explore alternative inflammasome pathways and the influence of metabolic factors, such as insulin, on inflammasome regulation in PCOS.

## 1. Introduction

Polycystic ovary syndrome (PCOS) is a prevalent endocrine disorder affecting 6–15% of women of reproductive age and is characterized by hyperandrogenism, oligo/anovulation, and polycystic ovarian morphology [1]. Beyond its reproductive features, PCOS is strongly associated with metabolic disturbances such as insulin resistance, obesity, and chronic low-grade inflammation [2]. These metabolic complications suggest that PCOS extends beyond the ovary, implicating peripheral tissues such as adipose tissue in its pathogenesis [3].

Adipose tissue, commonly referred to as body fat, is a type of loose connective tissue composed of three major components: adipocytes, the stromal vascular fraction (SVF), and the extracellular matrix (ECM) that surrounds the cells [4]. Adipocytes, which make up more than 80% of the tissue’s volume, are the primary cells responsible for energy storage and hormone secretion. The SVF includes a diverse group of cells such as immune cells (e.g., macrophages), preadipocytes, fibroblasts, vascular cells, and adipose-derived mesenchymal stem cells (ADSCs). The ECM provides structural support and consists mainly of collagen fibers, blood vessels, and nerve fibers [4].

Adipose tissue is now recognised as a key player in metabolic regulation and inflammation. In the context of obesity and insulin resistance, adipose tissue undergoes remodeling marked by immune cell infiltration and secretion of pro-inflammatory cytokines, such as IL-1β and IL-18 [5]. These cytokines contribute to systemic inflammation and impair insulin signalling pathways. Recent studies have emphasised that adipose tissue inflammation is not merely a consequence of obesity but may be a driving factor in the development of PCOS-related insulin resistance [5,6].

At the core of this inflammatory cascade is the NLRP3 inflammasome, a multiprotein complex comprising NLRP3, the adaptor protein ASC (PYCARD), and caspase-1 (CASP-1) [7]. Upon sensing metabolic danger signals such as reactive oxygen species or lipotoxicity, the inflammasome activates caspase-1, which in turn processes pro-IL-1β and pro-IL-18 into their mature, bioactive forms [7]. These cytokines are known to interfere with insulin receptor signalling and glucose metabolism, thereby contributing to systemic insulin resistance [7,8].

Strong evidence suggests that the NLRP3 inflammasome is a key mediator of inflammation and insulin resistance (IR) associated with obesity [3,9,10]. Increased NLRP3 expression in both subcutaneous and visceral adipose tissues (SAT and VAT) has been positively linked to higher BMI and IR in individuals with obesity [11]. NLRP3 inflammasome inhibition has been found to reduce obesity-related inflammation and improve insulin sensitivity [12]. Obesity-associated dyslipidaemia and lipotoxicity may act as endogenous danger signals (DAMPs), triggering inflammasome activation [12]. Additionally, NLRP3 activation in macrophages can be driven by obesity-related stimuli such as elevated ceramide levels, mitochondrial dysfunction, reactive oxygen species (ROS), and extracellular ATP [13,14,15].

Despite growing recognition of the inflammasome’s role in metabolic diseases, its specific contribution to the pathophysiology of PCOS remains underexplored. Particularly, there is a lack of data comparing subcutaneous (SAT) and visceral adipose tissue (VAT) compartments in women with PCOS with respect to inflammasome gene expression and protein levels.

In this study, we aimed to investigate the expression of key inflammasome-related genes (NLRP3, IL-1β, CASP1, and PYCARD) and the protein expression of NLRP3 in SAT and VAT from women with PCOS and age- and BMI-matched non-PCOS women. We used quantitative PCR to assess gene expression and Western blotting to evaluate NLRP3 protein levels. By comparing both fat depots, we aimed to better understand the contribution of adipose tissue inflammation to PCOS pathogenesis and to explore whether the NLRP3 inflammasome represents a mechanistic link between hyperandrogenism, adipose dysfunction, and insulin resistance in this condition.

## 2. Results

Baseline characteristics of the study population are summarized in Table 1. The two groups were comparable in age and BMI, with no statistically significant differences observed between them. All participants were of reproductive age and had not used any hormonal medications in the three months preceding the study, nor did they have a history of endocrine disorders or hypertension. The PCOS group met the Rotterdam criteria for PCOS including irregular menstrual cycles, hyperandrogenaemia, and polycystic ovaries on ultrasound scan. while the non-PCOS controls were selected based on the presence of a normal menstrual cycle and hormonal profile.

### 2.1. Gene Expression Levels of Caspase-1, NLRP3, IL-1β, and PYCARD Genes

#### 2.1.1. Caspase-1 (CASP-1) Gene Expression

**Figure 1:**
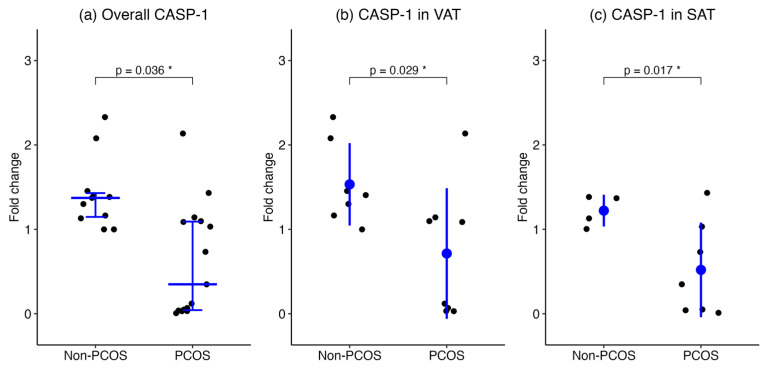
CASP-1 gene expression in adipose tissues from women with PCOS and BMI-matched controls. CASP-1 mRNA expression was assessed by qPCR in (**a**) combined SAT and VAT, (**b**) VAT, and (**c**) SAT. Data are presented as medians with interquartile ranges for overall group, and as mean ± SD for the VAT and SAT groups. Asterisks (*) indicate statistically significant differences (*p* < 0.05).

Overall, CASP-1 mRNA levels in SAT and VAT combined were significantly higher in non-PCOS participants (number of biopsies = 11) compared to those with PCOS (number of biopsies = 15) (median [IQR], 1.372 [1.131–1.454] vs. 0.3487 [0.03742–1.097], *p* = 0.036, respectively). When stratified by adipose depot, CASP-1 expression remained elevated in the non-PCOS group compared to the PCOS group in both VAT (number of biopsies = 7 vs. number of biopsies = 8; mean ± SD, 1.533 ± 0.4882 vs. 0.7139 ± 0.7738, *p* = 0.029, respectively) and SAT (number of biopsies = 4 vs. number of biopsies = 7; 1.222 ± 0.1883 vs. 0.5198 ± 0.5609, *p* = 0.017, respectively). These results suggest reduced CASP-1 transcriptional activity in both VAT and SAT among women with PCOS (Figure 1).

#### 2.1.2. NLRP3 Gene Expression

**Figure 2:**
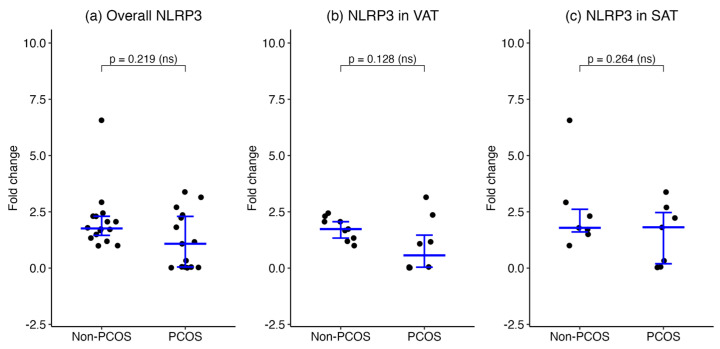
NLRP3 gene expression in adipose tissues of women with and without PCOS. NLRP3 mRNA expression was assessed by qPCR in (**a**) combined SAT and VAT, (**b**) VAT, and (**c**) SAT. Data are presented as medians with interquartile ranges for overall, VAT and SAT groups.

Overall, NLRP3 mRNA levels in SAT and VAT combined were not significantly different between the PCOS group (number of biopsies = 15) and the non-PCOS group (number of biopsies = 16) (median [IQR], 1.080 [0.04714–2.360] vs. 1.761 [1.374–2.305], *p* = 0.219, respectively). When stratified by adipose depot, NLRP3 expression in VAT tended to be lower in women with PCOS (number of biopsies = 8) compared to non-PCOS controls (number of biopsies = 9), although this difference did not reach statistical significance (median [IQR], 0.5657 [0.02518–2.062] vs. 1.733 [1.262–2.184], *p* = 0.128, respectively). In SAT, NLRP3 expression was comparable between the PCOS (number of biopsies = 7) and non-PCOS (number of biopsies = 7) groups (median [IQR], 1.814 [0.05709–2.703] vs. 1.789 [1.494–2.927], *p* = 0.264, respectively). These results indicate no significant difference in NLRP3 transcriptional activity between women with and without PCOS in either adipose depot (Figure 2).

#### 2.1.3. IL-1β Gene Expression

**Figure 3:**
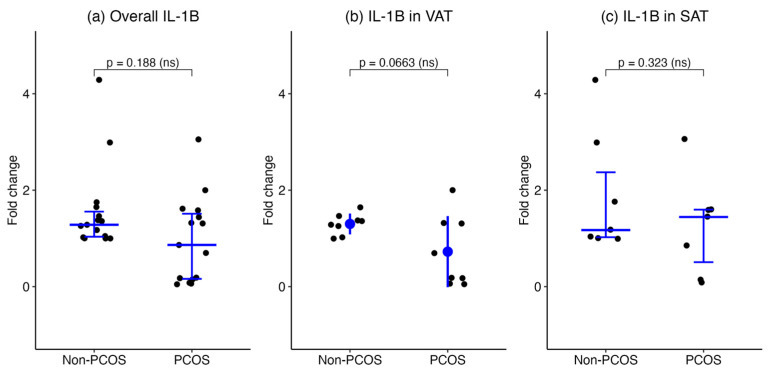
IL-1β gene expression in adipose tissues of women with and without PCOS. IL-1β mRNA expression was assessed by qPCR in (**a**) combined SAT and VAT, (**b**) VAT, and (**c**) SAT. Data are presented as medians with interquartile ranges for overall and SAT groups, and as mean ± SD for the VAT group.

Overall, IL-1β mRNA levels in SAT and VAT combined were not significantly different between the PCOS (number of biopsies = 15) and non-PCOS (number of biopsies = 15) groups (median [IQR], 0.8645 [0.1497–1.580] vs. 1.283 [1.021–1.647], *p* = 0.188, respectively). When stratified by adipose depot, IL-1β expression in VAT showed a non-significant trend toward lower levels in the PCOS group compared to non-PCOS controls (number of biopsies = 8 per group; mean ± SD, 0.7252 ± 0.7376 vs. 1.301 ± 0.2159, *p* = 0.066, respectively). In SAT, IL-1β expression was comparable between PCOS (number of biopsies = 7) and non-PCOS (number of biopsies = 7) groups (median [IQR], 1.444 [0.1497–1.613] vs. 1.173 [1.000–2.990], *p* = 0.323, respectively). These findings indicate that IL-1β gene expression in adipose tissue does not significantly differ by PCOS status (Figure 3).

#### 2.1.4. PYCARD Gene Expression

**Figure 4:**
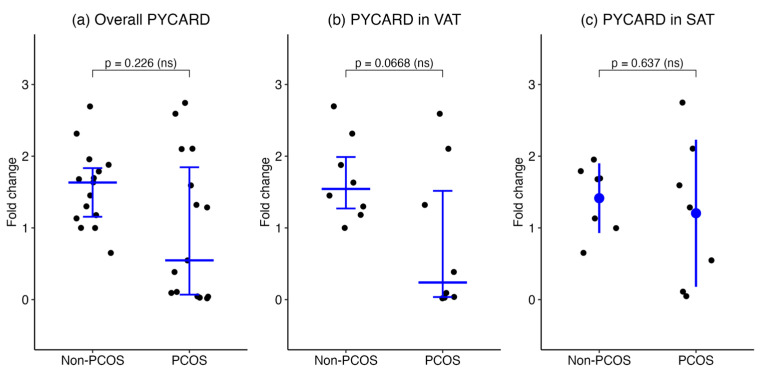
PYCARD gene expression in adipose tissue of women with PCOS and non-PCOS controls. PYCARD mRNA expression was assessed by qPCR in (**a**) combined SAT and VAT, (**b**) VAT, and (**c**) SAT. Data are presented as medians with interquartile ranges for overall and VAT groups and as mean ± SD for the SAT group.

Overall, PYCARD mRNA levels in SAT and VAT combined were lower in the PCOS group (number of biopsies = 15) compared to the non-PCOS group (number of biopsies = 15), although the difference was not statistically significant (median [IQR], 0.5471 [0.04671–2.101] vs. 1.633 [1.132–1.880], *p* = 0.226, respectively). When stratified by adipose depot, PYCARD expression in VAT showed a non-significant trend toward reduced levels in women with PCOS (number of biopsies = 8) compared to non-PCOS controls (number of biopsies = 8) (median [IQR], 0.2384 [0.02886–1.906] vs. 1.544 [1.210–2.205], *p* = 0.066, respectively). In SAT, PYCARD expression was comparable between PCOS and non-PCOS groups (number of biopsies = 7 each), with mean ± SD values of 1.204 ± 1.026 and 1.414 ± 0.4858, respectively (*p* = 0.637). Variance did not differ significantly between groups. These results indicate that PYCARD expression in adipose tissue does not significantly differ between women with and without PCOS (Figure 4).

### 2.2. NLRP3 Protein Expression in Adipose Tissue of Women with and Without PCOS

**Figure 5:**
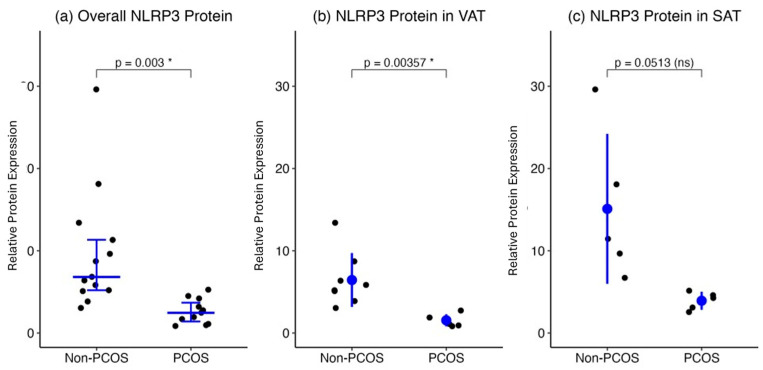
NLRP3 protein expression in adipose tissue from PCOS and non-PCOS women. NLRP3 protein levels were measured by Western blotting in (**a**) combined SAT and VAT, (**b**) visceral adipose tissue (VAT), and (**c**) subcutaneous adipose tissue (SAT). Data are presented as individual values with median ± interquartile range (IQR) for non-normally distributed data or mean ± SD for normally distributed data. Sample sizes are indicated in Section 4. Asterisks (*) indicate statistically significant differences (*p* < 0.05). Note that while the figures display all collected data points, the statistical analysis and reported *p*-values for the combined tissue (**a**) and stratified SAT (**c**) were calculated after the removal of one extreme outlier (value = 29.6) from the Non-PCOS SAT group.

Overall, NLRP3 protein levels in SAT and VAT combined were significantly (*p* = 0.003) higher in non-PCOS participants (number of biopsies = 13) compared to those with PCOS (number of biopsies = 10) (median [IQR], 6.813 [5.156–12.36] vs. 2.184 [1.056–4.267], respectively). When stratified by adipose depot, NLRP3 expression remained elevated in the non-PCOS group (number of biopsies = 8) compared to the PCOS group (number of biopsies = 6) in VAT (mean ± SD, 6.441 ± 3.281 vs. 1.543 ± 0.737, *p* = 0.0035, respectively). In SAT, although NLRP3 protein levels appeared higher in the non-PCOS group (number of biopsies = 5) than in the PCOS group (number of biopsies = 4) (15.10 ± 9.117 vs. 4.102 ± 1.184, *p* = 0.051, respectively), this difference did not reach statistical significance. These results suggest reduced NLRP3 protein abundance in adipose tissue, particularly in VAT, among women with PCOS (Figure 5).

After the removal of one extreme outlier (value = 29.6) from the non-PCOS group, Overall, NLRP3 protein levels in SAT and VAT combined were significantly higher in non-PCOS participants (number of biopsies = 12) compared to those with PCOS (number of biopsies = 10) (median [IQR]: 6.595 [5.156–11.22] vs. 2.184 [1.056–4.267], *p* = 0.004, respectively). When stratified by adipose depot, the removal of this outlier from the SAT data (Non-PCOS biopsies number = 4) resulted in a revised mean difference of 11.47± 4.812 vs. 4.102 ± 1.184, which was not statistically significant (*p* = 0.0510).

**Figure 6:**
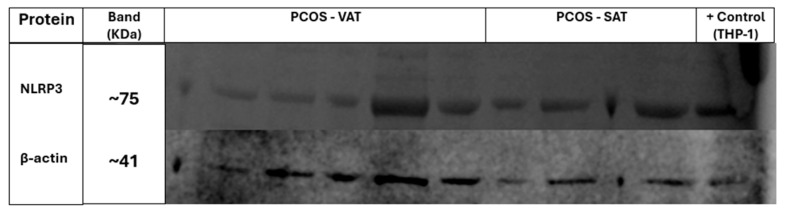
Western blot analysis of NLRP3 expression in adipose tissue from women with PCOS. Lanes 1–6 represent VAT, lanes 7–10 represent SAT, and lane 11 shows the positive control (THP-1 cell lysate). The upper panel shows NLRP3 protein bands at approximately 75 kDa, while the lower panel shows β-actin loading control bands at approximately 41 kDa.

**Figure 7:**
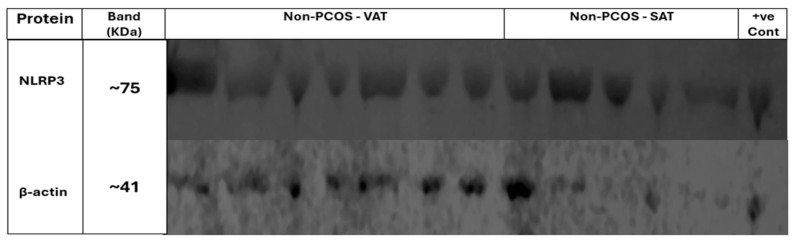
Western blot analysis of NLRP3 expression in adipose tissue from non-PCOS women. Lanes 1–8 represent VAT, lanes 9–13 represent SAT, and lane 14 shows the positive control (THP-1 cell lysate). The upper panel shows NLRP3 protein bands at approximately 75 kDa, while the lower panel shows β-actin loading control bands at approximately 41 kDa.

Western blot bands are shown for each group to illustrate NLRP3 expression in adipose tissue. Figure 6 displays NLRP3 (~75 kDa) and β-actin (~41 kDa) bands in samples from women with PCOS, while Figure 7 presents corresponding bands from non-PCOS participants. A strong NLRP3 signal is evident in several non-PCOS samples, particularly in VAT. The THP-1 cell lysate used as a positive control confirms antibody specificity with a clear NLRP3 band at ~75 kDa. β-actin served as a loading control in both blots to ensure equal protein loading. The original uncropped Western blot membranes for both Figure 6 and Figure 7 are available in Supplementary Information.

### 2.3. Plasma IL-1β and IL-18 Levels in PCOS and Non-PCOS Women

**Figure 8:**
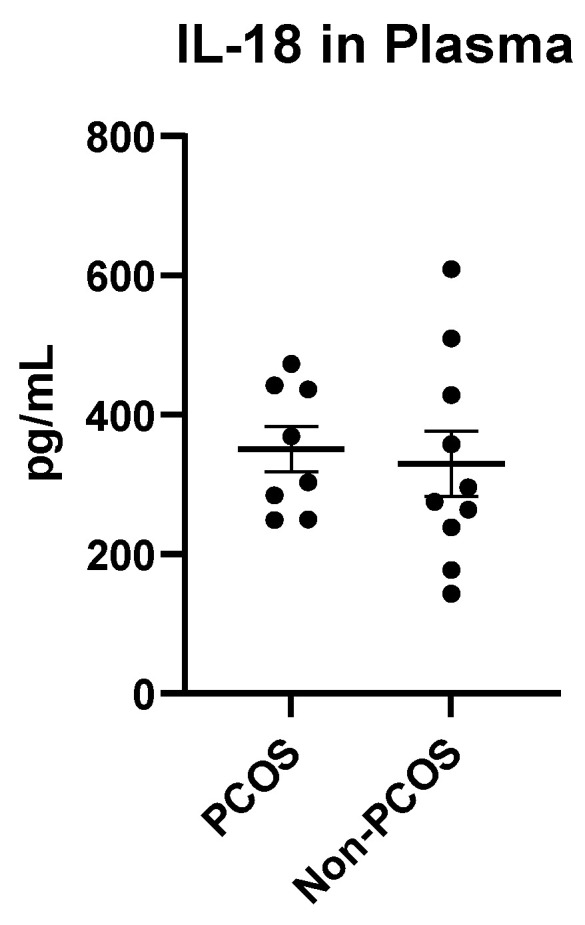
Plasma IL-18 levels in PCOS and non-PCOS women. Plasma IL-18 concentrations were measured by ELISA in samples from women with PCOS (n = 8) and non-PCOS controls (n = 10). Data are presented as mean ± SD. Statistical analysis using an unpaired *t*-test.

Plasma samples from women with PCOS (n = 8) and non-PCOS controls (n = 10) were analysed by ELISA to assess circulating levels of IL-1β and IL-18. IL-1β was undetectable in all plasma samples from both groups, indicating very low or absent systemic levels in this cohort. In contrast, IL-18 was detectable in plasma and exhibited measurable levels in both groups. Mean IL-18 concentration was 350.7 ± 91.23 pg/mL in the PCOS group and 329.8 ± 147.5 pg/mL in the non-PCOS group, with no statistically significant difference between them (*p* = 0.7175, unpaired *t*-test). These results confirm that while IL-1β is not present at detectable levels in circulation, IL-18 is consistently measurable and may serve as a useful positive control for inflammasome-related systemic activity (Figure 8).

## 3. Discussion

This study is the first to investigate the role of the NLRP3 inflammasome and its components, IL-1β, CASP-1, and PYCARD, in the pathogenesis of PCOS. We hypothesized that NLRP3 and its downstream effectors would be elevated in women with PCOS compared to healthy controls. Contrary to this hypothesis, CASP-1 gene expression was significantly higher in non-PCOS adipose tissue across VAT, SAT, and combined depots, while NLRP3 protein levels were reduced in VAT and combined samples, with no differences observed in SAT. Gene expression of NLRP3, IL-1β, and PYCARD did not differ between groups, suggesting a more complex or compensatory regulation of the inflammasome in PCOS. Overall, our findings provide no evidence of increased NLRP3 activity in PCOS and therefore do not support its direct role in PCOS pathogenesis. Nevertheless, due to potential technical and biological challenges, these results should be interpreted cautiously, and further well-designed studies are warranted to clarify these findings.

There have been no previous studies investigating NLRP3 in adipose tissue of women with PCOS. In a recent systematic review, we identified two human studies investigating NLRP3 in peripheral blood mononuclear cells (PBMCs), and one in luteinised granulosa cells (LGCs) obtained during IVF [3]. All three studies reported upregulation of NLRP3 and/or its components in PBMCs [16,17] and LGCs [18]. It is therefore surprising to observe lower NLRP3 expression in adipose tissue of women with PCOS compared with healthy control.

### 3.1. Chronic Inflammation in PCOS May Involve Other Inflammatory Pathways

While the NLRP3 inflammasome is a key player in chronic inflammation, our findings suggest it may not be the primary driver of immune activation in PCOS. This raises the possibility that other, alternative inflammasome pathways or caspase-independent mechanisms may contribute to PCOS-related inflammation. Future research should consider a broader immunological framework to identify additional pathways involved in the inflammatory profile of PCOS.

### 3.2. Chronic NLRP3 Activation and Potential Insulin-Mediated Suppression in PCOS

One compelling explanation for the lower NLRP3 expression observed in the PCOS group, despite the inflammatory nature of the condition, is the possibility of compensatory downregulation following chronic activation of the inflammasome. In PCOS, persistent metabolic stressors, such as insulin resistance, hyperinsulinemia, and elevated androgen levels, may chronically stimulate the NLRP3 inflammasome [16]. Over time, this ongoing stimulation could activate self-regulatory mechanisms designed to limit excessive inflammation and protect tissues from damage. As a result, the body may suppress NLRP3 expression as a negative feedback response, leading to reduced detectable levels even in the presence of inflammation. Supporting this, recent research has shown that insulin itself may play a direct anti-inflammatory role by interfering with NLRP3 activation [17]. Specifically, insulin has been found to reduce IL-1β secretion by blocking the formation of ASC specks and altering signalling pathways such as Syk and p38 MAPK [17]. In animal models, insulin administration lowered circulating IL-1β and reduced signs of inflammation in tissue [18,19]. Given that many women with PCOS exhibit chronically elevated insulin levels, it is plausible that insulin contributes to a dampening of the NLRP3 pathway in adipose tissue. This internal suppression mechanism, driven by both chronic inflammasome activation and insulin-mediated regulation, may help explain why PCOS samples showed lower NLRP3 expression compared to some control individuals, who might have experienced more acute or unregulated inflammatory signals. These findings highlight the importance of considering long-term metabolic adaptations when interpreting inflammatory marker expression and suggest that future research should further explore how insulin and chronic stimulation shape inflammasome activity in PCOS.

### 3.3. Possible Underlying Inflammation in Control Subjects

The unexpectedly higher expression of some inflammasome components in control subjects may reflect undiagnosed or subclinical inflammatory conditions. Chronic gynaecological issues such as pelvic inflammatory disease (PID) or endometriosis can persist without obvious clinical symptoms and may still influence inflammatory signalling pathways [20]. Although we took care to exclude participants with known inflammatory or autoimmune disorders, some conditions may have gone undetected. More comprehensive screening, such as measuring inflammatory biomarkers like C-reactive protein (CRP) or conducting pelvic imaging, could help ensure a more well-characterized and inflammation-free control group in future studies.

### 3.4. Detection of Truncated NLRP3 Protein Isoforms

The molecular weight of full-length NLRP3 is generally reported to range from 100 to ~118 kDa. However, several studies have observed alternative isoforms of different sizes. For example, Kummer et al. (2007) and Saresella et al. (2016) identified a truncated NLRP3 protein of approximately 75 kDa in human tissues, suggesting the presence of shorter isoforms [21,22]. Similarly, the datasheet for a commercial anti-NLRP3 antibody (Sigma-Aldrich, Cat. No. ABF23) notes that multiple isoforms ranging from 84 to 118 kDa have been observed in different lysates. Interestingly, a study investigating UVB-induced inflammasome activation in human keratinocytes, reported NLRP3 expression at ~140 kDa [23], further highlighting the variability in its apparent molecular weight across cell types and experimental conditions. Although we did not use the ABF23 antibody or assess keratinocytes, these findings support our detection of NLRP3 at ~75–80 kDa in adipose tissue as a plausible isoform or truncated variant. The biological role of this lower molecular weight form, particularly in the context of PCOS-related inflammation, remains to be elucidated and warrants further investigation.

### 3.5. mRNA-Protein Discrepancy

The discrepancy in expression levels between the mRNA and protein for NLRP3 in PCOS versus non-PCOS adipose tissue requires careful interpretation. This lack of correlation may be partly due to the limited number of samples available for the Western blot analysis. Furthermore, highly variable protein half-lives, which can range from mere seconds to several days, present an alternative explanation, potentially linked to differences in protein stability, post-translational modifications, or underlying RNA-silencing mechanisms [24,25,26].

### 3.6. Technical Challenges with Adipose Tissue

Working with adipose tissue presents unique technical challenges due to its fatty composition and vulnerability to degradation [27,28]. These factors can compromise both RNA and protein extraction, reducing yield and possibly impacting the reliability of downstream analyses like qPCR and Western blotting [27,28,29]. Additionally, adipose tissue varies in its cellular composition, vascularity, and fibrotic content, which can introduce further variability between samples [28,29]. It is possible that these technical issues hidden more consistent biological differences between groups. Future studies should standardize tissue handling protocols, from collection to processing, to minimize degradation and improve reproducibility. This may include using RNA stabilizing agents immediately post-sampling, optimized lysis buffers tailored to fatty tissues, and rapid freezing techniques.

### 3.7. Limitations

The sample size was relatively small, which may limit the generalizability of our findings and reduce the statistical power to detect minor differences. Larger cohorts are needed to validate and strengthen the observed associations. Secondly, using surgical patients as controls may have introduced confounding inflammation due to undiagnosed conditions potentially affecting inflammasome expression levels. Thirdly, the complex nature of adipose tissue, which is composed of multiple cell types, presents inherent technical challenges for precise molecular analysis, which must be considered when interpreting expression data. Consequently, the inability to assess changes in immune cell proportions, such as M1 vs. M2 macrophage polarisation, represents a limitation, as these shifts could be a major confounding factor influencing the total measured NLRP3/CASP-1 signal. Additionally, analysing adipose tissue alone may not fully represent systemic inflammasome dynamics, as other relevant tissues involved in PCOS pathophysiology, such as ovarian or immune cells, were not assessed.

Furthermore, we acknowledge technical limitations regarding the Western blot loading controls. In some instances, theβ-actin signal in the non-PCOS group exhibited lower visual clarity, which may introduce variability during the normalisation of target protein intensity. While all quantification was conducted using high-resolution digital scans to ensure the most robust data possible, this technical constraint warrants a cautious interpretation of the translational differences observed between the groups. Finally, the identification of truncated NLRP3 isoforms by Western blot requires further validation using complementary techniques to confirm their identity and functional relevance.

### 3.8. Future Directions

Future studies should broaden the scope beyond NLRP3 to explore other inflammatory and metabolic pathways that may contribute to PCOS, including alternative inflammasome mechanisms and caspase-independent processes. Given the potential regulatory role of insulin, further research is needed to investigate how insulin resistance and hyperinsulinemia influence inflammasome activity in adipose and other relevant tissues. Critically, future research must also investigate the immune cell composition of the adipose tissue, specifically whether shifts in M1 versus M2 macrophage populations contribute to the observed inflammasome dynamics. This should be achieved through analysis of specific macrophage markers and the use of histological staining to visualise and confirm the presence and distribution of immune cells in the different adipose depots. To improve reliability, future research should also address the technical limitations of adipose tissue handling by standardizing collection, processing, and preservation protocols. Additionally, refining control group selection by avoiding surgical patients and screening for subclinical inflammation will help reduce confounding factors. The biological relevance of truncated NLRP3 isoforms observed in this study should be further investigated using advanced protein characterization methods. Finally, studies incorporating ovarian tissue, as well as longitudinal and interventional designs, could help clarify the role and regulation of inflammasomes over time and in response to treatment in women with PCOS.

## 4. Materials and Methods

### 4.1. Study Subjects

All participants gave their informed consent, and the study was conducted in accordance with the Declaration of Helsinki and approved by the Health Research Authority (HRA) and Health and Care Research Wales (HCRW) (Research Ethics Committee reference: 21/EM/0282). The sponsor was the University of Nottingham, United Kingdom. Participants were divided into two groups: women diagnosed with PCOS according to the Rotterdam criteria [30] and non-PCOS healthy controls. Neither group included pregnant individuals. The control group included women with regular menstrual cycles (28–32-day cycle), normal serum levels of androgen and luteinizing hormone. Both groups were of childbearing age, ranging from 19–40 years with a BMI of ≤40 kg/m^2^. All participants had not taken any relevant medications for at least three months prior to the study. This included oral contraceptives, glucocorticoids, ovulation-inducing agents, anti-obesity drugs, anti-androgenic treatments, or antihypertensive medications. Women with endocrine disorders or hypertension were excluded.

#### 4.1.1. Sample Collection

##### Adipose Tissue Collection

Subcutaneous and visceral adipose tissue (~5 g) were obtained from both PCOS and control participants during elective surgical procedures. Immediately post-excision, tissues were transferred to the laboratory in a transport medium composed of Hanks’ Balanced Salt Solution (HBSS) supplemented with 1% penicillin (100 U/mL), streptomycin (100 U/mL), and 15 mM HEPES. In the lab, tissue samples were sectioned, weighed, and aliquoted into pre-labelled tubes, then snap-frozen in liquid nitrogen and stored at –80 °C for downstream molecular analyses. To preserve RNA integrity, strict RNase-free protocols were followed throughout the collection and handling process. This included the use of RNase Away™, RNase-free materials, and personal protective equipment (gloves and face masks). All tissue processing steps were performed within a Class II biosafety cabinet due to the potential biohazard risk.

##### Blood Collection

Venous blood samples were collected into EDTA tubes from each participant solely for plasma preparation. Plasma was promptly separated and stored at −80 °C until further analysis.

### 4.2. RNA Extraction and Quantitative PCR from Adipose Tissue

#### 4.2.1. RNA Isolation

Total RNA was extracted from 140–150 mg of frozen subcutaneous (SAT) and visceral (VAT) adipose tissue samples stored at –80 °C using a phenol-based method with QIAzol reagent (Qiagen, Valencia, CA, USA). Tissue was homogenized in 1 mL TRIzol using a Power Homogenizer (MP Biomedical, Solon, OH, USA) on ice. After a 5 min incubation at room temperature to dissociate nucleoprotein complexes, samples were centrifuged at 12,000× *g* for 15 min at 4 °C to remove the lipid layer. The clear lower phase was transferred, and phase separation was achieved by adding 0.2 mL of 1-Bromo-3-chloropropane per 1 mL of TRIzol, followed by gentle mixing and centrifugation. The upper aqueous phase, containing RNA, was carefully collected.

RNA was precipitated and further purified using the RNeasy Mini Kit (Qiagen, Valencia, CA, USA) with on-column DNase I treatment to eliminate genomic DNA contamination. The RNA pellet was washed with 70% ethanol and eluted in 30 μL RNase-free water. RNA purity and concentration were measured using a Nanodrop spectrophotometer (Thermo Fisher Scientific, Wilmington, DE, USA). Only samples with A260/A280 ratios between 1.98 and 2.30 were used for downstream analysis.

#### 4.2.2. cDNA Synthesis

cDNA was synthesized using the GoScript™ Reverse Transcription System (Promega, Madison, WI, USA). For each sample, 8 µL of total RNA was combined with 1 µL of Oligo(dT)15 primer, 1 µL of random primers, and nuclease-free water to a final volume of 10 µL. After heating at 70 °C for 5 min and immediate cooling on ice, a reverse transcription master mix (10 µL) was added containing reverse transcription buffer, MgCl_2_, dNTPs, RNase inhibitor, and GoScript™ reverse transcriptase. Reverse transcription was performed at 25 °C for 5 min, 42 °C for 45 min, and 70 °C for 15 min. Resulting cDNA was stored at –20 °C.

#### 4.2.3. Quantitative PCR (qPCR)

qPCR was performed using GoTaq Probe qPCR Master Mix (Promega, Madison, WI, USA) in 20 µL reactions comprising 10 µL of master mix, 1 µL of primer-probe mix, 4 µL nuclease-free water, and 5 µL of diluted cDNA. Reactions were run in technical triplicates on a QuantStudio 5 thermal cycler (Applied Biosystems, Foster, CA, USA) with the following conditions: 95 °C for 10 min, followed by 50 cycles of 95 °C for 15 s and 60 °C for 60 s.

Target genes included NLRP3, IL1β, CASP1, and PYCARD (ASC). Expression levels were normalised to a panel of candidate reference genes (PPIA, B2M, ACTB, and TFRC). The most stable reference gene, which was identified as PPIA using the NormFinder algorithm, was selected for normalisation. Relative gene expression was calculated using the ΔΔ*Ct* method. The Δ*Ct* value for each target gene was first determined by subtracting the *C_t_* value of PPIA from the Ct value of the target gene (Δ*Ct* = target *Ct* − PPIA *Ct*). The ΔΔ*Ct* value was then calculated by subtracting the average Δ*Ct* of the control samples from the Δ*Ct* of each target sample (ΔΔ*Ct* = Δ*Ct* target − Δ*Ct* control Average). The final Fold Change was subsequently derived using the 2^−ΔΔ*Ct*^ formula [31]. Assay specificity was confirmed by melt curve analysis, and assay efficiencies were determined using standard curves from serial cDNA dilutions.

### 4.3. Protein Quantification and Western Blotting

Total protein was extracted from 200–300 mg with RIPA lysis buffer (Cat. P0013C, Beyotime Biotechnology, Shanghai, China). Protein concentrations were determined using the Bicinchoninic Acid (BCA) Protein Assay Kit (Thermo Fisher, Cat# 23225, Waltham, MA, USA), following the manufacturer’s instructions. A standard curve was generated using serial dilutions of Bovine Serum Albumin (BSA) ranging from 0 to 2000 µg/mL. In a 96-well flat-bottom plate, 25 µL of each standard and sample (in duplicate) was combined with 200 µL of freshly prepared BCA working reagent (WR; 50:1 ratio of Reagent A to Reagent B). After incubation at room temperature for 30 min, absorbance was read at 560 nm using an Omega microplate reader (Tecan Austria GmbH, Grödig, Austria). Protein concentrations were interpolated from the standard curve.

For Western blot analysis, equal amounts of protein were mixed with LDS sample buffer and BOLT reducing agent, brought to a final volume of 20 µL with deionised water, then heated at 100 °C for 5–10 min. Samples were cooled on ice, briefly centrifuged, and loaded (20 µL) onto pre-cast SDS-PAGE gels alongside 9 µL of protein ladder. Protein lysates from THP-1 monocytes were included as a positive control for NLRP3 expression. Electrophoresis was carried out at 200 V for 35 min.

Proteins were transferred onto nitrocellulose membranes using a wet transfer system at 10 V for 90 min in transfer buffer (prepared with 800 mL dH_2_O, 150 mL methanol, and 50 mL 20× transfer buffer). The gel-membrane sandwich was assembled with pre-soaked filter paper and sponges, and air bubbles were removed using a roller. Membranes were stained with Ponceau S to confirm transfer, rinsed with Dh2o, and then blocked overnight at 4 °C in PBS-T containing 3% non-fat dry milk.

Following blocking, membranes were incubated for 4 h at 4 °C with primary antibodies diluted in blocking buffer: NLRP3 (D4D8T) Rabbit mAb (#15101, Cell Signalling Technology, Danvers, MA, USA; 1:500), with β-Actin (Abcam™, Cambridge, UK, ab8226; 1:1000), used as a loading control. After three 5 min washes in PBS-T, membranes were incubated for 1 h at room temperature with IRDye^®^ secondary antibodies: Donkey anti-rabbit IgG (IRDye® 800CW, #926-32213, Lincoln, NE, USA) and Donkey anti-mouse IgG (IRDye^®^ 680RD, #926-68072, Lincoln, NE, USA), both at 1:10,000 dilution. After final washes, membranes were imaged using the LI-COR Odyssey system, and signal intensities were quantified using Image Studio™ software (version 4.1). All membranes were analysed under identical exposure conditions to ensure consistency. Protein expression was normalised to β-actin.

### 4.4. Quantification of Plasma IL-1β and IL-18 by ELISA

Plasma levels of interleukin-1β (IL-1β) and interleukin-18 (IL-18) were measured using DuoSet ELISA kits from R&D Systems (Minneapolis, MN, USA) (IL-1β: DY201; IL-18: DY31805), following the manufacturer’s recommended protocol.

96-well microplates were coated overnight at room temperature with 100 µL/well of the capture antibody diluted in phosphate-buffered saline (PBS) without carrier protein. The next day, plates were washed three times with 400 µL/well of wash buffer and then blocked with 300 µL/well of reagent diluent (PBS containing 1% BSA) for 1 h at room temperature. After blocking, the plates were washed again as before.

Standard curves were generated using seven serial dilutions: IL-1β standards ranged from 250 pg/mL to 3.91 pg/mL, and IL-18 standards from 750 pg/mL to 11.7 pg/mL. Plasma samples were added to the plate undiluted. All standards and samples were added at 100 µL/well in duplicate. Plates were covered and incubated for 2 h at room temperature, followed by another wash step. Next, 100 µL/well of biotinylated detection antibody, diluted in reagent diluent, was added and incubated for 2 h at room temperature.

After washing, 100 µL/well of streptavidin–horseradish peroxidase (HRP) was added and incubated for 20 min at room temperature in the dark. Following a final wash step, 100 µL of substrate solution was added to each well and incubated for 20 min at room temperature. The reaction was stopped by adding 50 µL of stop solution per well.

Optical density was measured immediately at 450 nm using a microplate reader, with wavelength correction at 570 nm to account for plate imperfections. IL-18 was used as a positive control to validate assay performance. Cytokine concentrations were calculated from standard curves generated with recombinant standards included in the kits.

### 4.5. Statistical Analysis

Statistical analyses for quantitative real-time PCR (qPCR) and Western blotting were performed using R statistical software version 2025.09.2-418 (R Foundation for Statistical Computing, Vienna, Austria). GraphPad Prism version 10.4.0 (GraphPad Software, San Diego, CA, USA) was used for the ELISA analysis. Each experiment was repeated at least three times for each participant sample to ensure data reliability and reproducibility. Prior to statistical testing, outlier detection was applied to all datasets using the ROUT method (Q = 1%); any identified outliers were subsequently reported in the results. Normality of each data subset was then assessed using the Shapiro–Wilk test.

To account for the paired nature of VAT and SAT samples from the same individual, a Two-Way Mixed-Effects Model (ANOVA) was conducted for qPCR and Western blotting data. The overall difference between PCOS and non-PCOS groups was determined by the Main Effect of Group from this model. For tissue-specific comparison (PCOS VAT vs. non-PCOS VAT, or PCOS SAT vs. non-PCOS SAT), post hoc pairwise comparisons were derived from the Mixed-Effects Model.

For the ELISA data, where samples were independent, the statistical test was chosen based on data distribution: the parametric unpaired *t*-test was applied for normally distributed data, and the non-parametric Mann–Whitney U test was applied for non-normally distributed data.

Data visualisation was generated using R and GraphPad Prism. For tissue-specific data and overall, values are presented as means and standard deviation (SD) if a parametric test was used, or as medians with interquartile ranges (IQR) if a non-parametric test was used [32]. A *p*-value of <0.05 was considered statistically significant.

## 5. Conclusions

Our study did not find evidence of any increase in NLRP3 inflammasome activity in women with PCOS compared to healthy controls. While CASP-1 gene expression and NLRP3 protein levels were lower in adipose tissue from PCOS participants, gene expression of NLRP3, IL-1β, and PYCARD were comparable between the two groups. These results suggest that the role of the NLRP3 inflammasome in PCOS is more complex than previously assumed. The unexpected lack of any increased NLRP3 activity may be due to compensatory regulatory mechanisms or other metabolic influences such as insulin. Given the limitations of the current study, including sample size and technical challenges in adipose tissue analysis, these findings should be interpreted with caution. Further well-designed studies are required to confirm these observations, explore other inflammasome pathways, and clarify how metabolic factors modulate inflammasome activity in PCOS.

## Figures and Tables

**Table 1:** Demographic and clinical characteristics of 18 study participants.

Parameters	Non-PCOS (n = 10)	PCOS (n = 8)	*p*
Age (yrs)	29.0 (19–40)	26.9 (21–38)	0.389
BMI (kg/m^2^)	30.0 (25–34)	31.3 (29–34.5)	0.228
Ethnicity	90.9% WB, 9.1% PNS	90.9% WB, 9.1% PNS	
Regular menstrual cycles (%)	RC: 100%	RC: 27.3%, O: 54.5%, Am: 9.1%, IRC:9.1%	
LH (IU/L)	4.806 ± 3.807	10.42 ± 6.533	0.03
Testosterone (nmol/L)	0.929 ± 0.570	2.01 ± 0.795	0.005
SHBG (nmol/L)	46.40 [42.53–66.15]	22.10 [12.85–36.95]	<0.001
FAI	1.83 ± 1.145	10.07 ± 6.026	0.003

Data are summarised as mean (range), mean ± SD, or median [interquartile range]. LH: Luteinising Hormone; SHBG: Sex Hormone-Binding Globulin; FAI: Free Androgen Index; WB: White British; PNS: Prefer Not to Say; RC: Regular menstrual cycle; IRC: Irregular menstrual cycle; O: Oligomenorrhoea; Am: Amenorrhoea. Note: The study included 10 women in the control group and 8 women in the PCOS group (a total of 18 participants). While the participant numbers are fixed, the exact number of tissue samples available for each molecular analysis (e.g., qPCR, Western blot) varied due to tissue yield and quality. Therefore, the sample size for all specific assays in Section 2 is reported using the convention (number of biopsies = ) to ensure accurate representation of the tissues analysed.

## Data Availability

The data supporting the findings of this study are available from the corresponding author upon reasonable request. The data are not publicly available due to ethical and privacy restrictions related to human participants.

## Supplementary Materials

The following supporting information can be downloaded at: https://www.mdpi.com/article/10.3390/ijms27020699/s1.
