# Supplementary material for: NLRP3 Inflammasome and Polycystic Ovary Syndrome (PCOS): A Novel Profile in Adipose Tissue
Source: Int J Mol Sci. 2026 Jan 9;27(2):699. doi: 10.3390/ijms27020699 (PMC12840877; doi:10.3390/ijms27020699)

Supplementary:

Figure 6:

- **Uncropped β-actin only image (PCOS):**

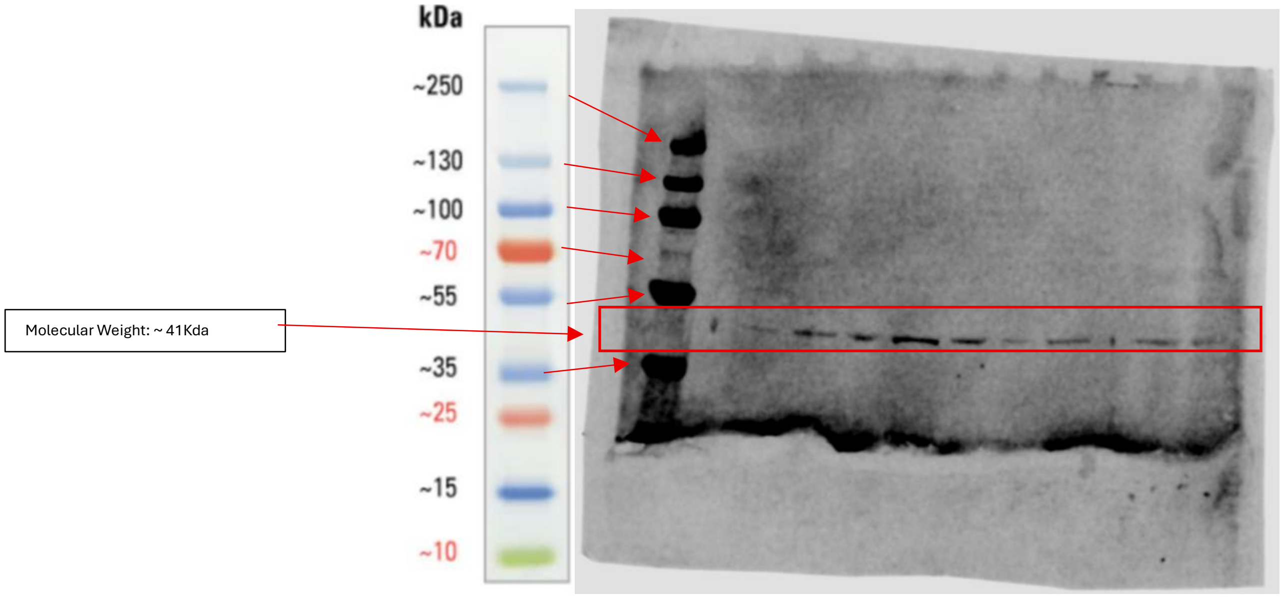

In this figure, I showed only the β-actin membrane because I wanted to demonstrate that the loading control was clean and reliable. When we developed the membrane, we closed the chamber for the protein of interest (NLRP3) so that only the β-actin chamber remained open. By doing this, the β-actin bands appear clearly, without any signal from the protein of interest. This helps show that:

1. The loading was equal across all lanes.
2. The exposure for β-actin is not affected by the other antibody.
3. The figure is easier to interpret because only one antibody signal is visible.

Figure 6:

- **Uncropped NLRP3 protein only image (PCOS):**

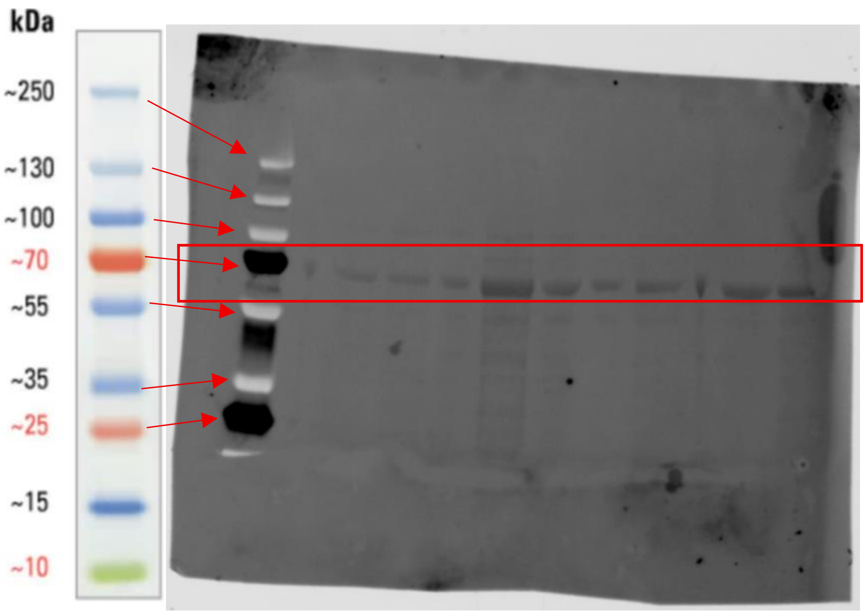

In this figure, I showed only the protein of interest (NLRP3), I kept the β-actin chamber closed. This allowed to show a clear image of the target protein without any interference from the loading control. Displaying the protein alone is useful because:

1. It shows the specific band pattern for the NLRP3 protein.
2. There is no background from β-actin, so the bands are easier to interpret.
3. It helps confirm that any differences we see are due to changes in the protein, not the loading control.

Figure 6:

- **Uncropped NLRP3 & Beta actin image (PCOS):**

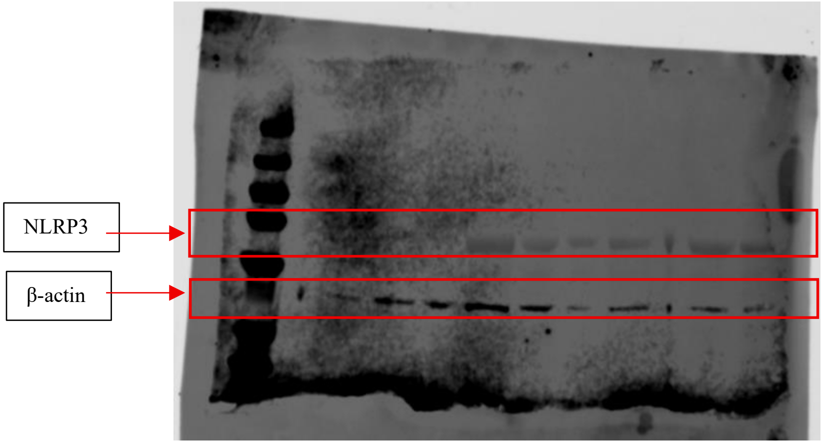

In this figure, both chambers (NLRP3 + β-actin) were opened, the membrane showed signals from both antibodies at the same time. This made the picture look messy because:

1. The protein of interest bands and the β-actin band were developed together.
2. The two signals can overlap or increase the background, especially if the exposures are different.
3. The final image becomes less clear, making it harder to see the quality of each band separately.

Figure 7:

- ## Uncropped β-actin & NLRP3/β-actin images (non-PCOS):

Due to the challenge of achieving optimal exposure for all components in a single image, Figure A is included to ensure clearer correspondence of the β-actin band to the molecular weight ladder. Figure B displays the composite, uncropped image used for densitometry, which contains the clearest representation of both ~ 41 kDa loading control bands and the corresponding NLRP3 bands across all samples

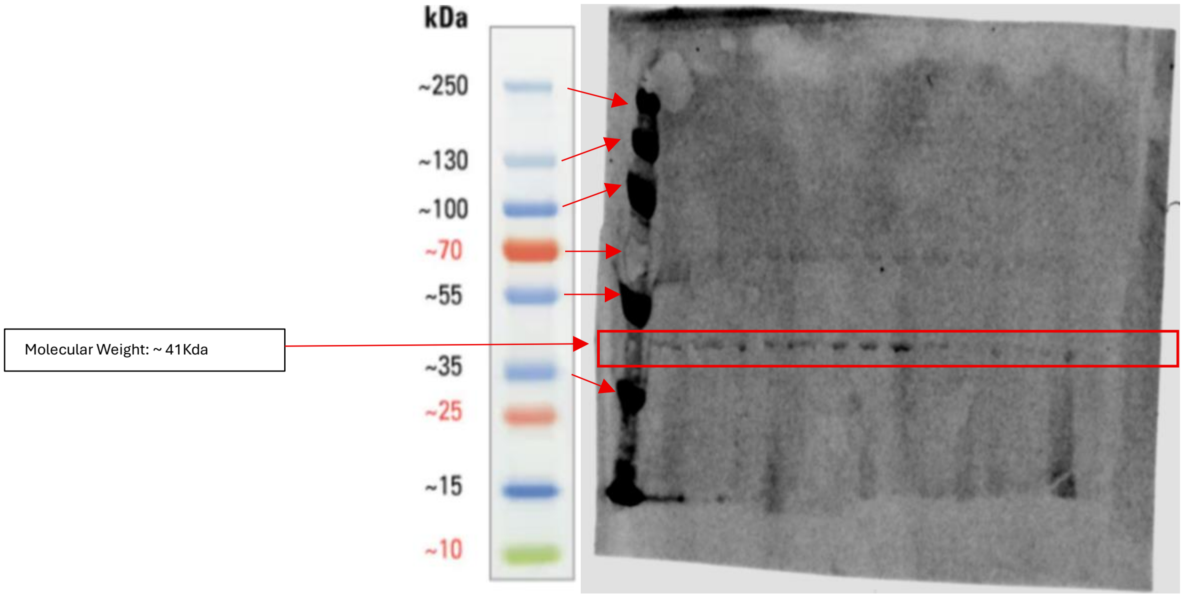

Fig. A

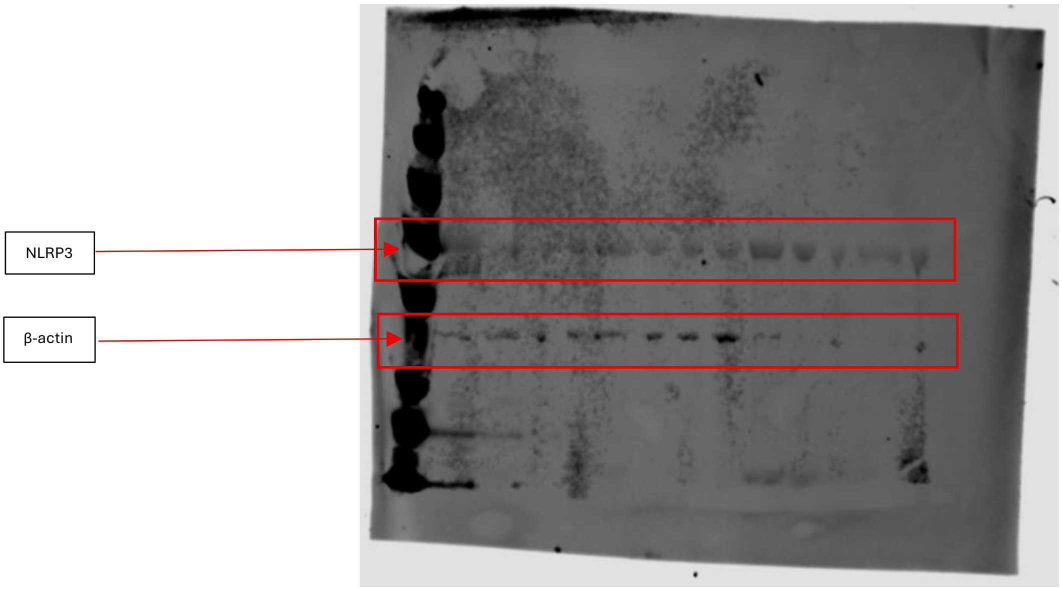

Fig. B

Figure 7:

- **Uncropped NLRP3 only image (non-PCOS):**

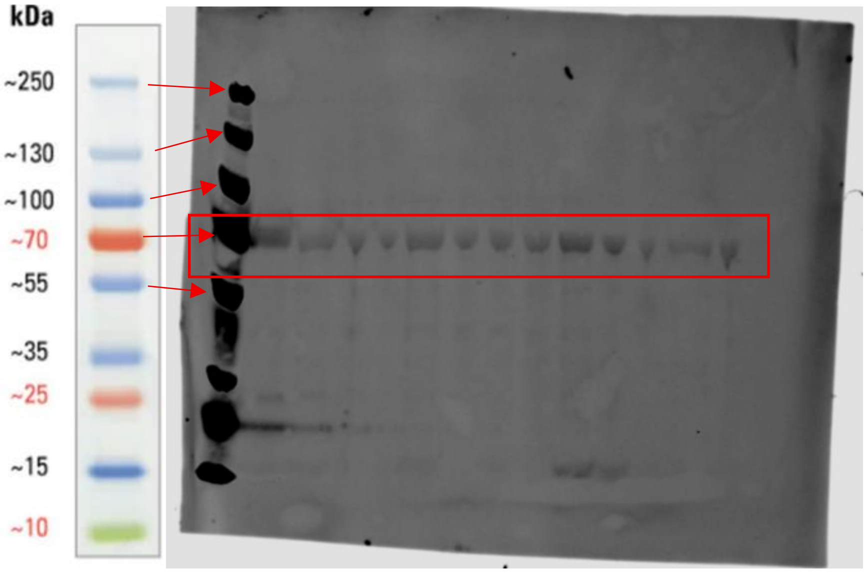

Supplement: Supplementary file 1 [file ijms-27-00699-s001.zip › ijms-4003513-supplementary.pdf]
